# Supplementary material for: Optimal strategies for learning multi-ancestry polygenic scores vary across traits
Source: Nat Commun. 2023 Jul 7;14:4023. doi: 10.1038/s41467-023-38930-7 (PMC10328935; doi:10.1038/s41467-023-38930-7)
Supplement: Supplementary file 1 — Supplementary Information [file 41467_2023_38930_MOESM1_ESM.pdf]

Supplementary Material for 'Optimal strategies for learning multi-ancestry polygenic scores vary across traits'

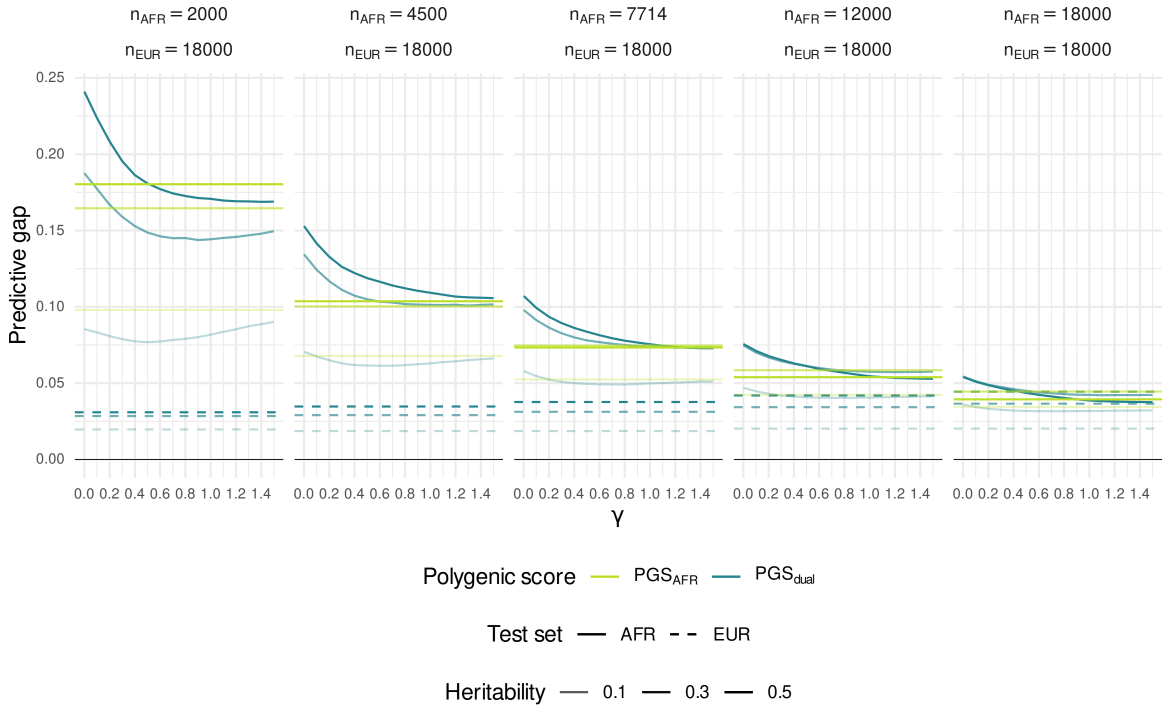

Supplementary Figure 1: **Supplementary simulation study: predictive gap against number of African-ancestry individuals in training set.** Each panel corresponds to a different number of African-ancestry training set individuals from $n_{AFR} = 2\,000$ to $n_{AFR} = 18\,000$. The training sets for $\mathrm{PGS}_{\mathrm{dual}}$ (blue lines) consisted of the corresponding African-ancestry training set for $\mathrm{PGS}_{\mathrm{AFR}}$ (yellow lines), along with $n_{EUR} = 18\,000$ European-ancestry individuals. The γ parameter represents the degree of reweighting in the dual-ancestry training set, with $\gamma = 0$ corresponding to no reweighting, and $\gamma = 1$ corresponding to inverse proportion reweighting (see Materials and Methods for details). Each line represents the mean predictive gap across 50 repetitions. The horizontal dashed lines correspond to the predictive gap (the difference between the optimal r^2 and the observed r^2 for a PGS) for European-ancestry test sets based on an unweighted LASSO, while the solid lines correspond to the predictive gap for African-ancestry test sets. The correlation of genetic effects between ancestries ρ was fixed at 0.8. The number of causal SNPs p_0 was fixed at 100. The overall heritability was varied from 0.1 (lighter lines) to 0.5 (darker lines).

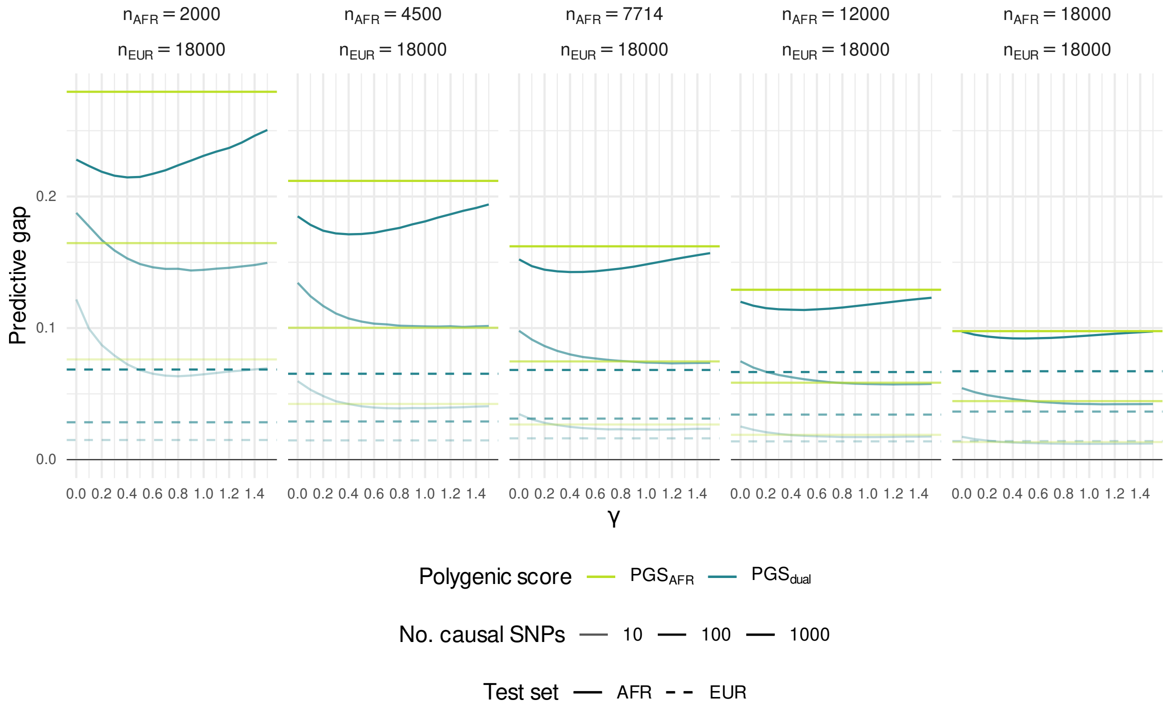

Supplementary Figure 2: **Supplementary simulation study: predictive gap against number of African-ancestry individuals in training set.** Each panel corresponds to a different number of African-ancestry training set individuals from $n_{AFR} = 2\,000$ to $n_{AFR} = 18\,000$. The training sets for PGS_{dual} (blue lines) consisted of the corresponding African-ancestry training set for PGS_{AFR} (yellow lines), along with $n_{EUR} = 18\,000$ European-ancestry individuals. Each line represents the mean predictive gap across 50 repetitions. The horizontal dashed lines correspond to the predictive gap for European-ancestry test sets based on an unweighted LASSO, while the solid lines correspond to the predictive gap for African-ancestry test sets. The correlation of genetic effects between ancestries ρ was fixed at 0.8. The overall heritability h_2 was fixed at 0.3. The number of causal SNPs was varied from 10 (lighter lines) to 1000 (darker lines).

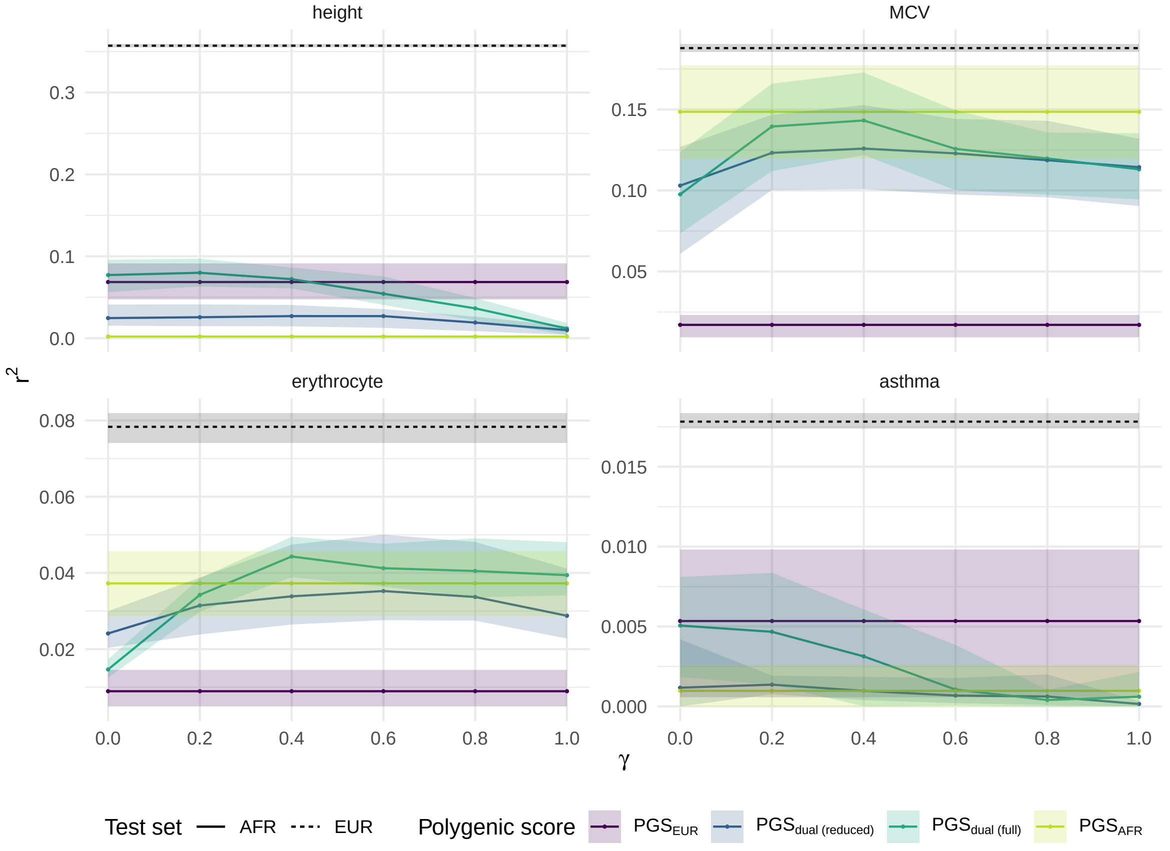

Supplementary Figure 3: **Predictive performance of dual-ancestry PGS constructed using all available European-ancestry and African-ancestry individuals.** The dashed lines correspond to predictive performance on a European-ancestry test set using PGS_{EUR}. Predictive performance on an African-ancestry test set is shown by the solid lines. The single-ancestry scores were estimated using a standard, unweighted LASSO. The dual-ancestry scores were constructed using an importance weighted LASSO with various degrees of reweighting γ (see Materials and Methods). Error bars correspond to the range across five cross-validation rounds of training set construction and PGS estimation. The four traits considered are height, mean corpuscular volume (MCV), asthma, and erythrocyte distribution width. $\text{PGS}_{\text{dual (full)}}$ was trained on the union of the African-ancestry and European-ancestry training sets, while $\text{PGS}_{\text{dual (reduced)}}$ was made up of the African-ancestry training set combined with European-ancestry individuals so that the proportion of African-ancestry individuals was 10%.

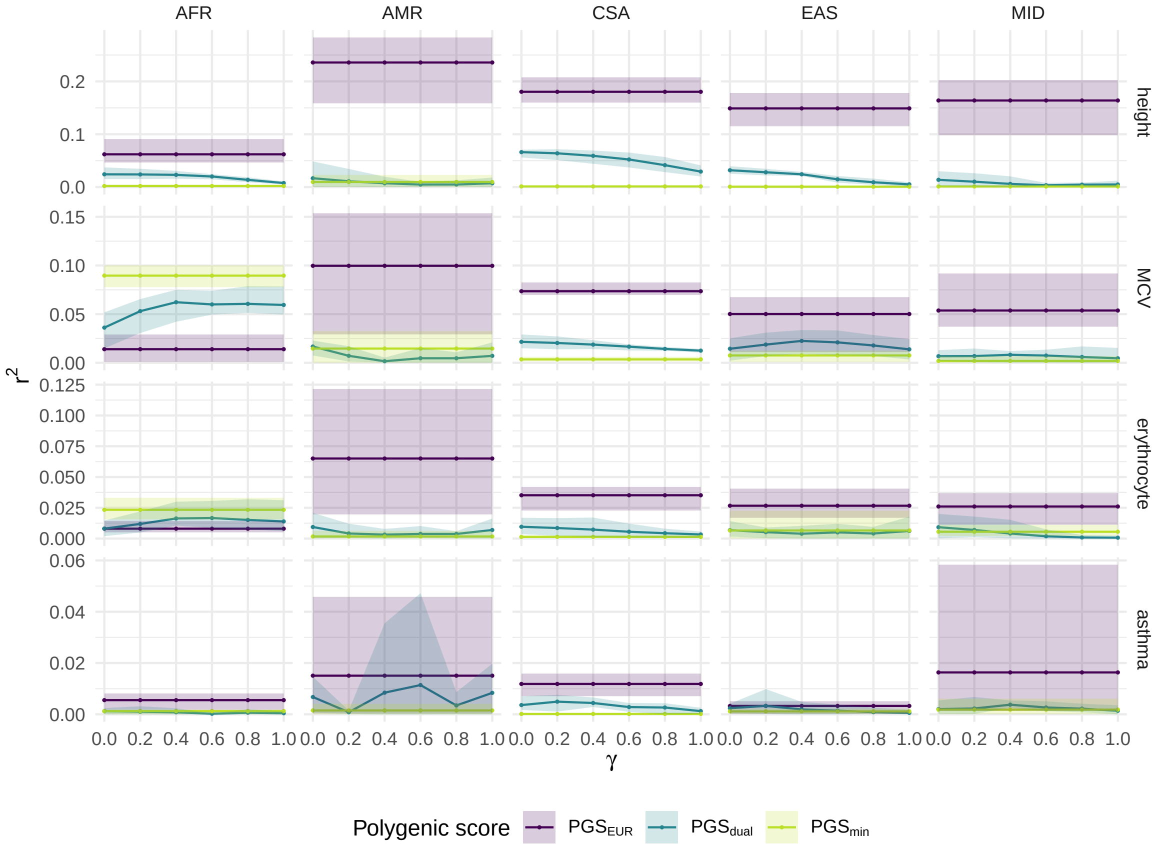

Supplementary Figure 4: **Partial/pseudo r^2 for European-, multi-, and minority-ancestry PGS on four traits in UK Biobank for five minority-ancestry groups using genotyped SNPs only.** The single-ancestry scores were estimated using a standard, unweighted LASSO. The dual-ancestry scores were constructed using an importance weighted LASSO with various degrees of reweighting γ (see Materials and Methods). Error bars correspond to the range across five cross-validation rounds of training set construction and PGS estimation. The four traits considered are height, mean corpuscular volume (MCV), asthma, and erythrocyte distribution width. We used inferred genetic ancestry labels from Pan-UKBB, with participants divided into six groups: European ancestry (EUR), African ancestry (AFR), Admixed American ancestry (AMR), Central/South Asian ancestry (CSA), East Asian ancestry (EAS), and Middle Eastern ancestry (MID).

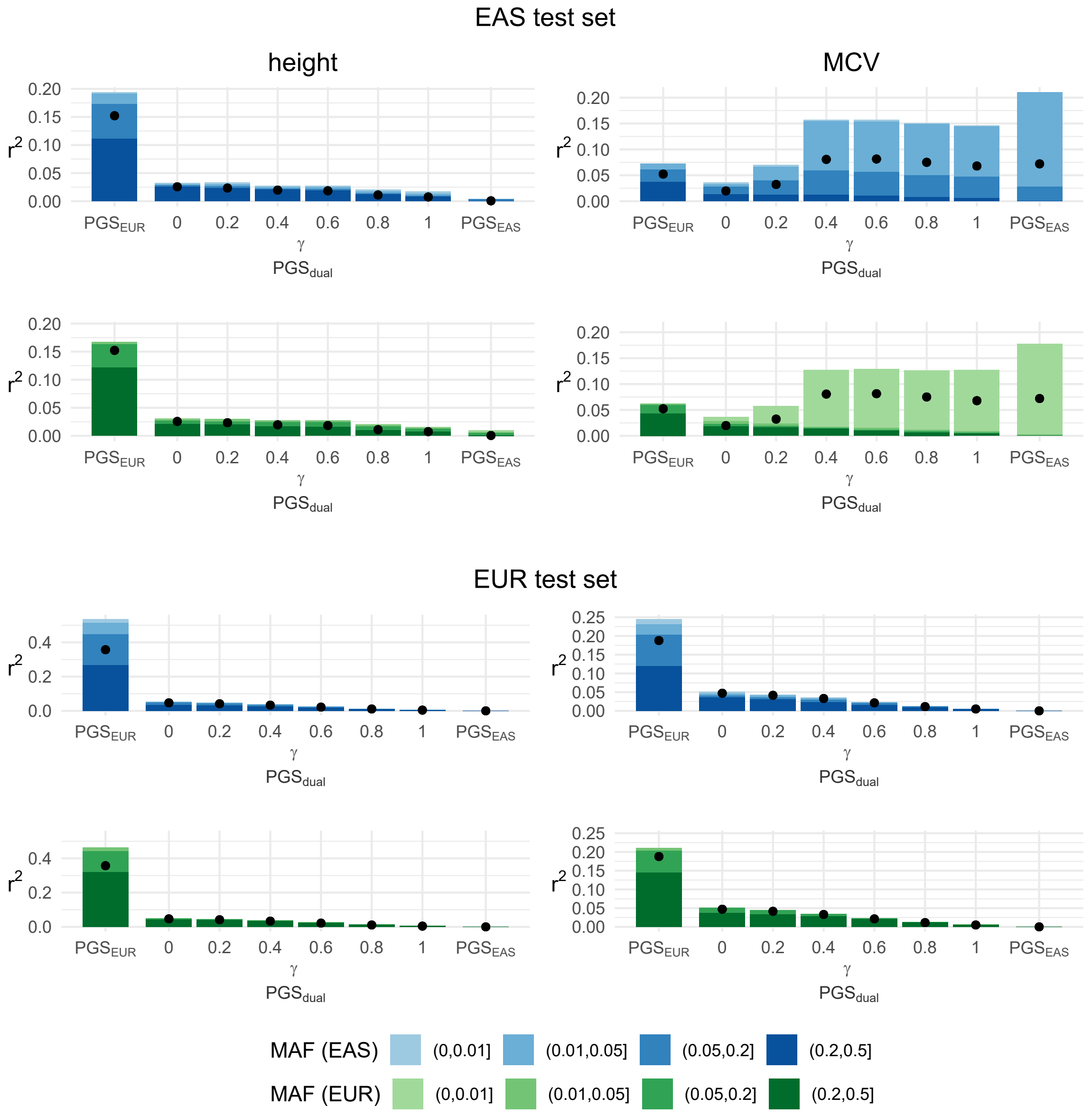

Supplementary Figure 5: Allele frequency composition of variance explained by PGS for height (left) and mean corpuscular volume (right) in an East Asian ancestry test set (top) and a European-ancestry test set (bottom), based on European-, multi-, and minority-ancestry PGS. The single-ancestry scores were estimated using a standard, unweighted LASSO. The dual-ancestry scores were constructed using an importance weighted LASSO with various degrees of reweighting γ (see Materials and Methods). The black dots represent partial r^2 for all the variants, i.e. the entire polygenic score. Variants were grouped according to their minor allele frequency in an East Asian ancestry individuals (blue palette) or in European-ancestry individuals (green palette). Each bar represents the sum of the partial r^2 values for each subset of variants in a given polygenic score. Note that the bars are stacked, and the height of the bar is generally higher than corresponding dot due to linkage disequilibrium between variants.

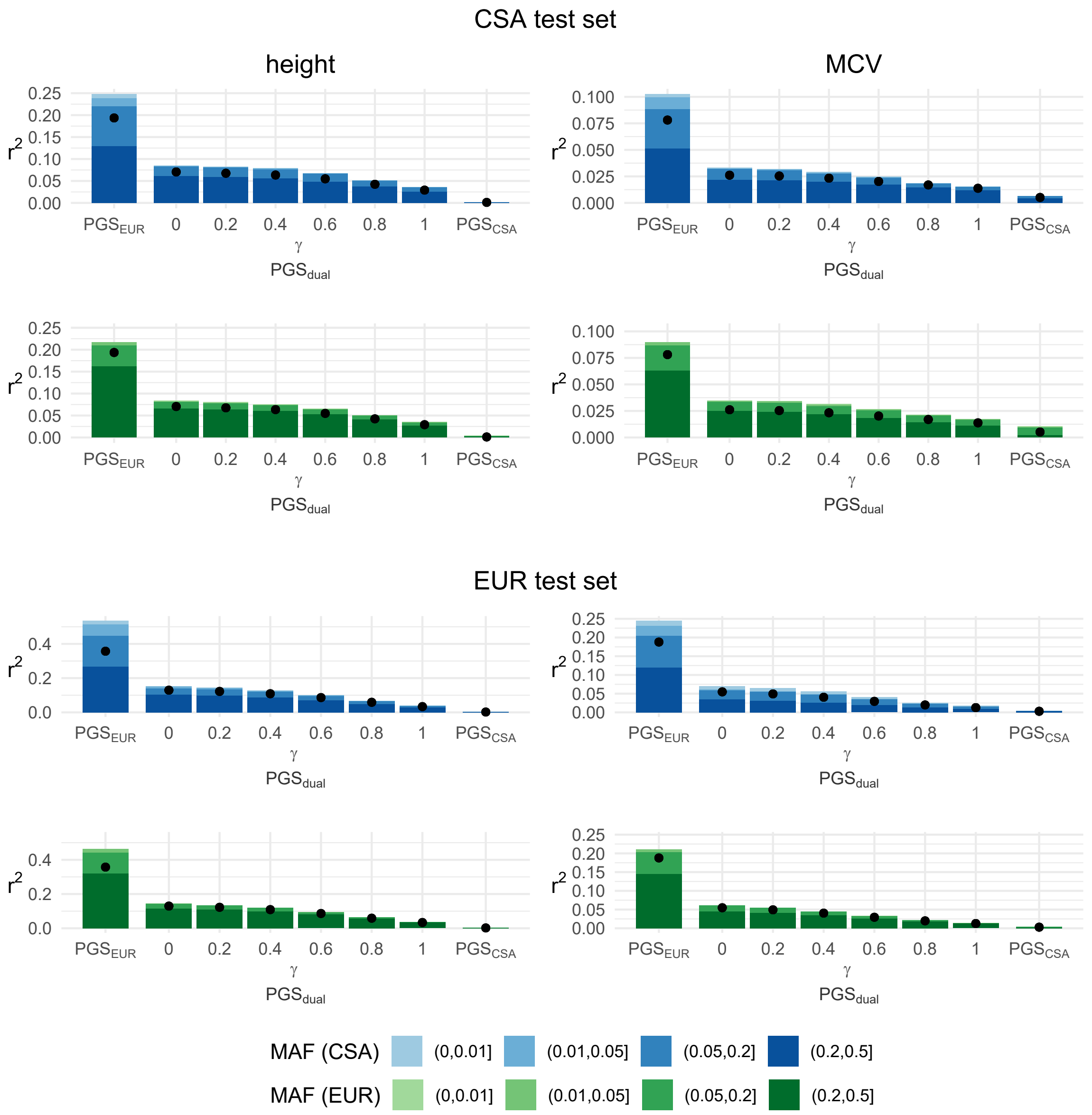

Supplementary Figure 6: Allele frequency composition of variance explained by PGS for height (left) and mean corpuscular volume (right) in a Central/South Asian ancestry test set (top) and a European-ancestry test set (bottom), based on European-, multi-, and minority-ancestry PGS. The single-ancestry scores were estimated using a standard, unweighted LASSO. The dual-ancestry scores were constructed using an importance weighted LASSO with various degrees of reweighting γ (see Materials and Methods). The black dots represent partial r^2 for all the variants, i.e. the entire polygenic score. Variants were grouped according to their minor allele frequency in a Central/South Asian ancestry individuals (blue palette) or in European-ancestry individuals (green palette). Each bar represents the sum of the partial r^2 values for each subset of variants in a given polygenic score. Note that the bars are stacked, and the height of the bar is generally higher than corresponding dot due to linkage disequilibrium between variants.

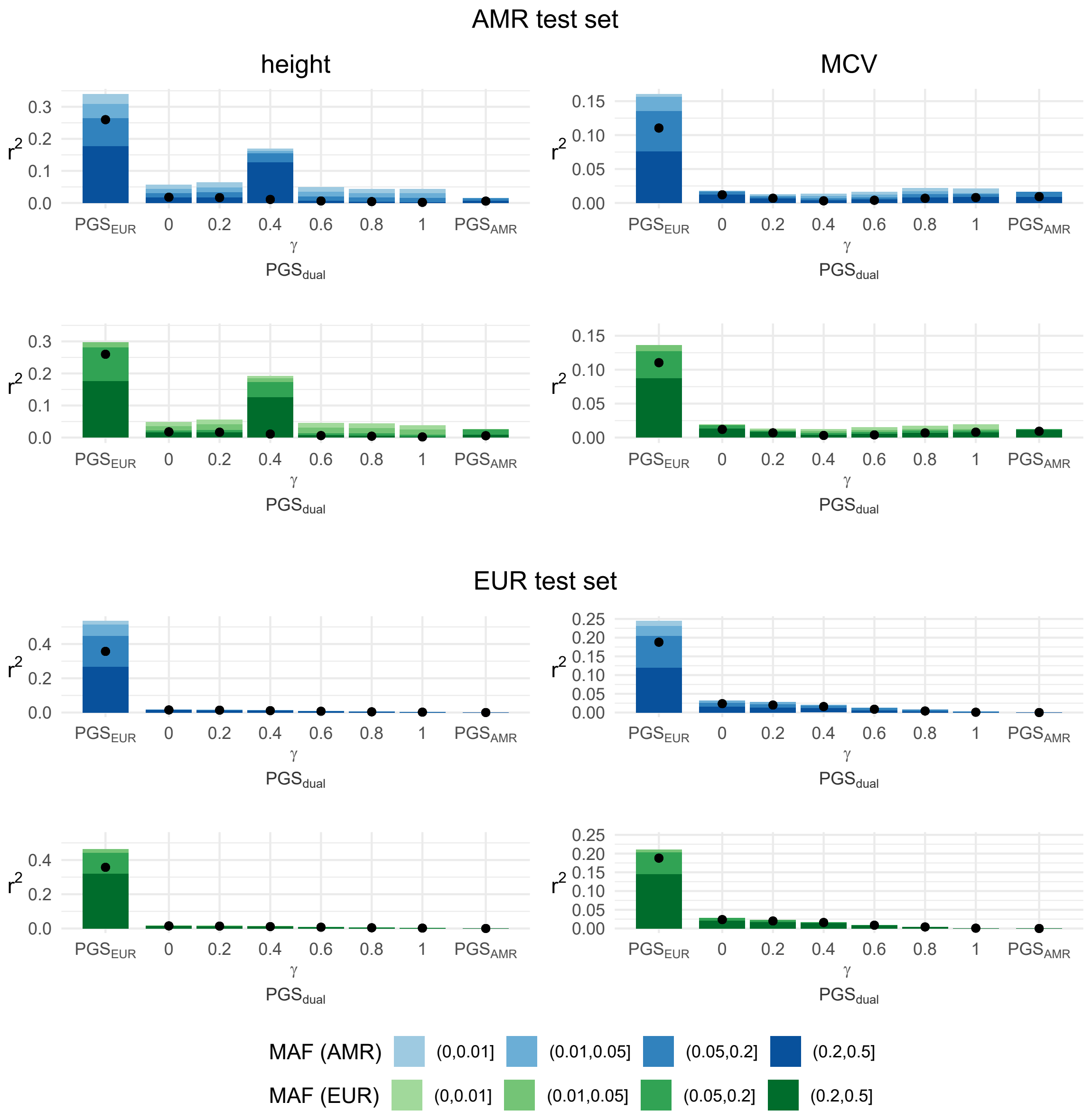

Supplementary Figure 7: Allele frequency composition of variance explained by PGS for height (left) and mean corpuscular volume (right) in an Admixed American ancestry test set (top) and a European-ancestry test set (bottom), based on European-, multi-, and minority-ancestry PGS. The single-ancestry scores were estimated using a standard, unweighted LASSO. The dual-ancestry scores were constructed using an importance weighted LASSO with various degrees of reweighting γ (see Materials and Methods). The black dots represent partial r^2 for all the variants, i.e. the entire polygenic score. Variants were grouped according to their minor allele frequency in an Admixed American ancestry individuals (blue palette) or in European-ancestry individuals (green palette). Each bar represents the sum of the partial r^2 values for each subset of variants in a given polygenic score. Note that the bars are stacked, and the height of the bar is generally higher than corresponding dot due to linkage disequilibrium between variants.

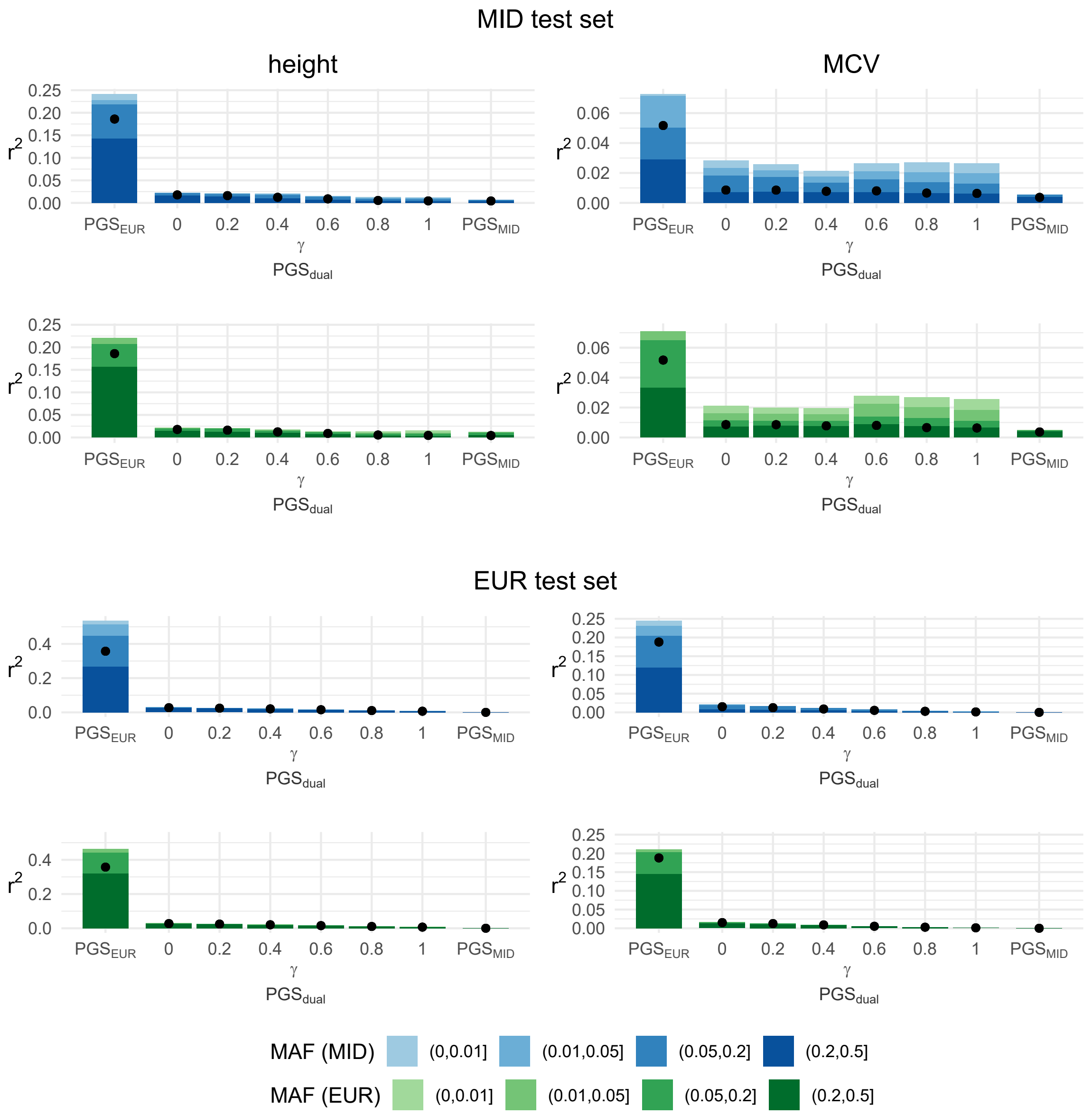

Supplementary Figure 8: Allele frequency composition of variance explained by PGS for height (left) and mean corpuscular volume (right) in a Middle Eastern ancestry test set (top) and a European-ancestry test set (bottom), based on European-, multi-, and minority-ancestry PGS. The single-ancestry scores were estimated using a standard, unweighted LASSO. The dual-ancestry scores were constructed using an importance weighted LASSO with various degrees of reweighting γ (see Materials and Methods). The black dots represent partial r^2 for all the variants, i.e. the entire polygenic score. Variants were grouped according to their minor allele frequency in a Middle Eastern ancestry individuals (blue palette) or in European-ancestry individuals (green palette). Each bar represents the sum of the partial r^2 values for each subset of variants in a given polygenic score. Note that the bars are stacked, and the height of the bar is generally higher than corresponding dot due to linkage disequilibrium between variants.

Supplementary Table 1: Relative variance of polygenic scores across traits and minority ancestry groups. Here, $\hat{g}_{EUR}^{min}$ denotes the vector of European-ancestry polygenic scores ($\mathrm{PGS}_{\mathrm{EUR}}$) for the minority-ancestry test set, while $\hat{g}_{min}^{EUR}$ denotes the vector of minority-ancestry polygenic scores ($\mathrm{PGS}_{\mathrm{min}}$) for the European-ancestry test set. Then, $\widetilde{\mathrm{Var}}(\hat{g}_{EUR}^{min})$ denotes the sample variance of $\hat{g}_{EUR}^{min}$ divided by the sample variance of $\hat{g}_{EUR}^{EUR}$. The minority ancestry labels correspond to the inferred genetic ancestry labels from Pan-UKBB, with participants divided into six groups: European ancestry (EUR), African ancestry (AFR), Admixed American ancestry (AMR), Central/South Asian ancestry (CSA), East Asian ancestry (EAS), and Middle Eastern ancestry (MID).

Trait	Minority ancestry	$\widetilde{\mathrm{Var}}(\hat{g}_{EUR}^{min})$	$\widetilde{\mathrm{Var}}(\hat{g}_{min}^{min})$	$\widetilde{\mathrm{Var}}(\hat{g}_{min}^{EUR})$
Atrial fibrillation (AFib)	AFR	0.799	18.327	13.263
Body mass index (BMI)	AFR	0.578	0.221	0.169
Diverticular disease of the intestine	AFR	0.688	3.766	2.935
Female genital prolapse	AFR	0.662	89.554	74.022
Mean corpuscular volume (MCV)	AFR	0.562	1.722	0.353
Mean corpuscular volume (MCV)	AMR	0.937	1.388	1.406
Mean corpuscular volume (MCV)	CSA	0.867	0.350	0.334
Mean corpuscular volume (MCV)	EAS	0.840	1.125	0.607
Mean corpuscular volume (MCV)	MID	0.884	0.720	0.674
Mean platelet volume	AFR	0.543	0.103	0.076
Asthma	AFR	0.617	2.313	1.762
Asthma	AMR	0.930	33.283	35.773
Asthma	CSA	0.904	4.303	4.096
Asthma	EAS	0.732	5.493	4.666
Asthma	MID	0.921	11.419	12.006
Eosinophill percentage	AFR	0.614	0.319	0.240
Erythrocyte distribution width	AFR	0.551	2.790	1.110
Erythrocyte distribution width	AMR	0.977	1.986	2.124
Erythrocyte distribution width	CSA	0.937	0.791	0.743
Erythrocyte distribution width	EAS	0.778	0.863	0.709
Erythrocyte distribution width	MID	0.937	1.680	1.643
Height	AFR	0.545	0.050	0.039
Height	AMR	0.928	0.406	0.392
Height	CSA	0.818	0.051	0.047
Height	EAS	0.672	0.105	0.084
Height	MID	0.901	0.554	0.577
Hypothyroidism	AFR	0.605	3.800	2.882
Lymphocyte count	AFR	0.615	0.563	0.423
Monocyte count	AFR	0.612	0.466	0.343
Platelet crit	AFR	0.609	0.283	0.225
High light scatter reticulocyte count	AFR	0.674	0.502	0.183
