# Supplementary material for: Optimal strategies for learning multi-ancestry polygenic scores vary across traits
Source: Nat Commun. 2023 Jul 7;14:4023. doi: 10.1038/s41467-023-38930-7 (PMC10328935; doi:10.1038/s41467-023-38930-7)
Supplement: Supplementary file 2 — Reporting Summary [file 41467_2023_38930_MOESM2_ESM.pdf]

nature portfolio

Corresponding author(s): Brieuc Lehmann

Last updated by author(s): Apr 28, 2023

Reporting Summary

Nature Portfolio wishes to improve the reproducibility of the work that we publish. This form provides structure for consistency and transparency in reporting. For further information on Nature Portfolio policies, see our Editorial Policies and the Editorial Policy Checklist.

Statistics

For all statistical analyses, confirm that the following items are present in the figure legend, table legend, main text, or Methods section.

n/a	Confirmed	
☐	☒	The exact sample size (*n*) for each experimental group/condition, given as a discrete number and unit of measurement
☐	☒	A statement on whether measurements were taken from distinct samples or whether the same sample was measured repeatedly
☒	☐	The statistical test(s) used AND whether they are one- or two-sided<br>*Only common tests should be described solely by name; describe more complex techniques in the Methods section.*
☐	☒	A description of all covariates tested
☐	☒	A description of any assumptions or corrections, such as tests of normality and adjustment for multiple comparisons
☐	☒	A full description of the statistical parameters including central tendency (e.g. means) or other basic estimates (e.g. regression coefficient) AND variation (e.g. standard deviation) or associated estimates of uncertainty (e.g. confidence intervals)
☒	☐	For null hypothesis testing, the test statistic (e.g. *F*, *t*, *r*) with confidence intervals, effect sizes, degrees of freedom and *P* value noted<br>*Give P values as exact values whenever suitable.*
☒	☐	For Bayesian analysis, information on the choice of priors and Markov chain Monte Carlo settings
☒	☐	For hierarchical and complex designs, identification of the appropriate level for tests and full reporting of outcomes
☒	☐	Estimates of effect sizes (e.g. Cohen's *d*, Pearson's *r*), indicating how they were calculated

Our web collection on statistics for biologists contains articles on many of the points above.

Software and code

Policy information about availability of computer code

Data collection	No software was used for data collection.
Data analysis	The code used to generate these results is available at https://github.com/brieuclehmann/pgs_transferability/releases/tag/v0.1. To construct the PGS, we used the glmnet R package v4.1 (for simulated data), and a slight adaptation of the snpnet R package v0.3.0 available at https://github.com/brieuclehmann/snpnet (for UK Biobank data). To generate simulated data, we used the msprime simulation engine via the stdpopsim v0.1.2 python library - see https://popsim-consortium.github.io/stdpopsim-docs/. To preprocess the UK Biobank genetic data, we made use of both PLINK v1.9 and PLINK v2.0, available at https://www.cog-genomics.org/plink/.

For manuscripts utilizing custom algorithms or software that are central to the research but not yet described in published literature, software must be made available to editors and reviewers. We strongly encourage code deposition in a community repository (e.g. GitHub). See the Nature Portfolio guidelines for submitting code & software for further information.

Data

Policy information about availability of data

All manuscripts must include a data availability statement. This statement should provide the following information, where applicable:

- Accession codes, unique identifiers, or web links for publicly available datasets
- A description of any restrictions on data availability
- For clinical datasets or third party data, please ensure that the statement adheres to our policy

This research has been conducted using data from UK Biobank, a major biomedical database, under application 12788. The UK Biobank data are available under restricted access; access can be obtained by researchers upon application - see https://www.ukbiobank.ac.uk/enable-your-research. The genetic correlation estimates from the Neale Lab UKBB Genetic Correlation Browser are available at https://ukbb-rg.hail.is/. The heritability estimates from the Neale Lab UKB SNP-Heritability Browser are available at https://nealelab.github.io/UKBB_ldsc/downloads.html. The ancestry-specific heritability estimates and inferred genetic ancestry labels for UK Biobank individuals from the Pan-UKBB initiative are available at are available at https://pan.ukbb.broadinstitute.org/downloads. The recombination map of GRCh37 is available as part of the stdpopsim python library - see https://popsim-consortium.github.io/stdpopsim-docs.

Research involving human participants, their data, or biological material

Policy information about studies with human participants or human data. See also policy information about sex, gender (identity/presentation), and sexual orientation and race, ethnicity and racism.

Reporting on sex and gender	Our study is methodological in nature and investigates the composition of training sets for the construction of polygenic scores in ancestrally diverse populations. As such, we did not perform sex- or gender-based analyses.
Reporting on race, ethnicity, or other socially relevant groupings	We used genetic ancestry labels inferred by the Pan-UKBB initiative (https://pan.ukbb.broadinstitute.org/). To control for population structure, we included the following covariates in our model: age, sex, the first ten genetic principal components (PCs), and interactions between sex and the ten genetic PCs.
Population characteristics	The UK Biobank dataset is described in PMID 25826379 and PMID 30305743. Participants were aged 40-69 years when recruited in 2006-2010. The UK Biobank genetic data contains genotypes for 488,377 participants.
Recruitment	No original recruitment was undertaken by this study.
Ethics oversight	UK Biobank has approval from the North West Multi-centre Research Ethics Committee (MREC) as a Research Tissue Bank (RTB) approval. This approval means that researchers do not require separate ethical clearance and can operate under the RTB approval.

Note that full information on the approval of the study protocol must also be provided in the manuscript.

Field-specific reporting

Please select the one below that is the best fit for your research. If you are not sure, read the appropriate sections before making your selection.

☒ Life sciences ☐ Behavioural & social sciences ☐ Ecological, evolutionary & environmental sciences

For a reference copy of the document with all sections, see nature.com/documents/nr-reporting-summary-flat.pdf

Life sciences study design

All studies must disclose on these points even when the disclosure is negative.

Sample size	No original data collection was performed in this study. We used available UK Biobank data, and one of the aims of our analyses was to assess the role of sample size in the performance of polygenic scoring. We investigated a range of quantitative and binary traits to support our analyses, selecting traits with at least 5% SNP heritability to ensure sufficient signal. We also did not run any analyses on binary trait-ancestry combinations for which the number of cases was less than 50, again to ensure sufficient signal.
Data exclusions	We removed individuals who requested to be removed from the UK Biobank study. We also removed individuals who were identified as displaying sex chromosome aneuploidy. We also removed individuals who were flagged as related by Pan-UKBB. Within each ancestry group, related individuals were identified using PC-Relate with k=10 and a minimum individual MAF of 5%. We filtered out variants that were not deemed to be of `high quality' according to Pan-UKBB, retaining those with an INFO score of at least 0.8 and with an allele count of at least 20 per population.
Replication	We repeated our analyses across five rounds of cross-validation to improve the robustness of our results. Our conclusions were supported even when accounting for the uncertainty afforded by these cross-validations rounds.
Randomization	Individuals within each ancestry group were randomised into five folds as part of the above cross-validation procedure.
Blinding	Blinding at the data analysis stage was ensured via the randomised cross-validation procedure - each of the cross-validation folds was analysed identically. No original data collection was carried out as part of this study.

Reporting for specific materials, systems and methods

We require information from authors about some types of materials, experimental systems and methods used in many studies. Here, indicate whether each material, system or method listed is relevant to your study. If you are not sure if a list item applies to your research, read the appropriate section before selecting a response.

Materials & experimental systems

n/a	Involved in the study
☒	☐ Antibodies
☒	☐ Eukaryotic cell lines
☒	☐ Palaeontology and archaeology
☒	☐ Animals and other organisms
☒	☐ Clinical data
☒	☐ Dual use research of concern
☒	☐ Plants

Methods

n/a	Involved in the study
☒	☐ ChIP-seq
☒	☐ Flow cytometry
☒	☐ MRI-based neuroimaging
